# First reported *Tannerella forsythia* infection in a patient with extensive bronchiectasis: a case report

**DOI:** 10.3389/fmed.2025.1571506

**Published:** 2025-04-28

**Authors:** Congli Fu, Yilan Sun, Chun Chen

**Affiliations:** Cancer Center, Department of Pulmonary and Critical Care Medicine, Zhejiang Provincial People's Hospital, Affiliated People's Hospital, Hangzhou Medical College, Hangzhou, China

**Keywords:** non-cystic fibrosis bronchiectasis, oral pathogen, *Tannerella forsythia*, mNGs, antibiotics

## Abstract

*Tannerella forsythia* infection was common in oral diseases but less reported in lung diseases. This report presents a patient with bronchiectasis who was infected by *Tannerella forsythia* and subsequently hospitalized with symptoms including fever, progressive cough, sputum production, and shortness of breath. A chest computed tomography (CT) scan revealed multiple bilateral pulmonary bronchiectasis with signs of infection. Metagenomic next-generation sequencing (mNGS) of the bronchoalveolar lavage fluid primarily detected *Tannerella forsythia*. Treatment with Piperacillin-tazobactam and ornidazole resulted in a favorable outcome. This case first reported a patient with extensive bronchiectasis infected by *Tannerella forsythia* and provided an effective treatment.

## Background

Bronchiectasis, a common pulmonary disease often characterized by inflammation, frequently presents with bacterial infections. In recent years, numerous studies have explored the bacteriome in bronchiectasis, identifying genera such as Pseudomonas, Haemophilus, Streptococcus, Veillonella, Prevotella, Stenotrophomonas, and Neisseria (1). Elderly patients with bronchiectasis often suffer from malnutrition and may experience weakened swallowing function. If these patients develop dental caries, oral pathogens can easily invade the lungs. However, the role of oral pathogens in bronchiectasis has received limited attention. *Tannerella forsythia*, a gram-negative bacterium, typically does not thrive aerobically. Instead, it is commonly associated with odontogenic diseases such as chronic periodontitis, gingivitis, and aggressive periodontitis. This article represents the first documented case of *Tannerella forsythia* infection in a patient with bronchiectasis.

## Case presentation

A 70-year-old male was admitted to the hospital presenting with severe cough, chest pressure, and a low-grade fever. He had been experiencing symptoms of cough, sputum production, and shortness of breath following physical activity for over 40 years. About a week prior to admission, he noticed a worsening of these symptoms accompanied by fever, prompting him to seek treatment at a local community hospital. Despite receiving antibiotics such as cefuroxime axetil tablets and cough medication, his symptoms persisted. The patient denied any episodes of hemoptysis or chest pain. Subsequently, he was referred to our hospital for further evaluation. The patient had worked as a quarry owner for nearly four decades and reported no history of smoking or alcohol consumption.

**Figure 1:**
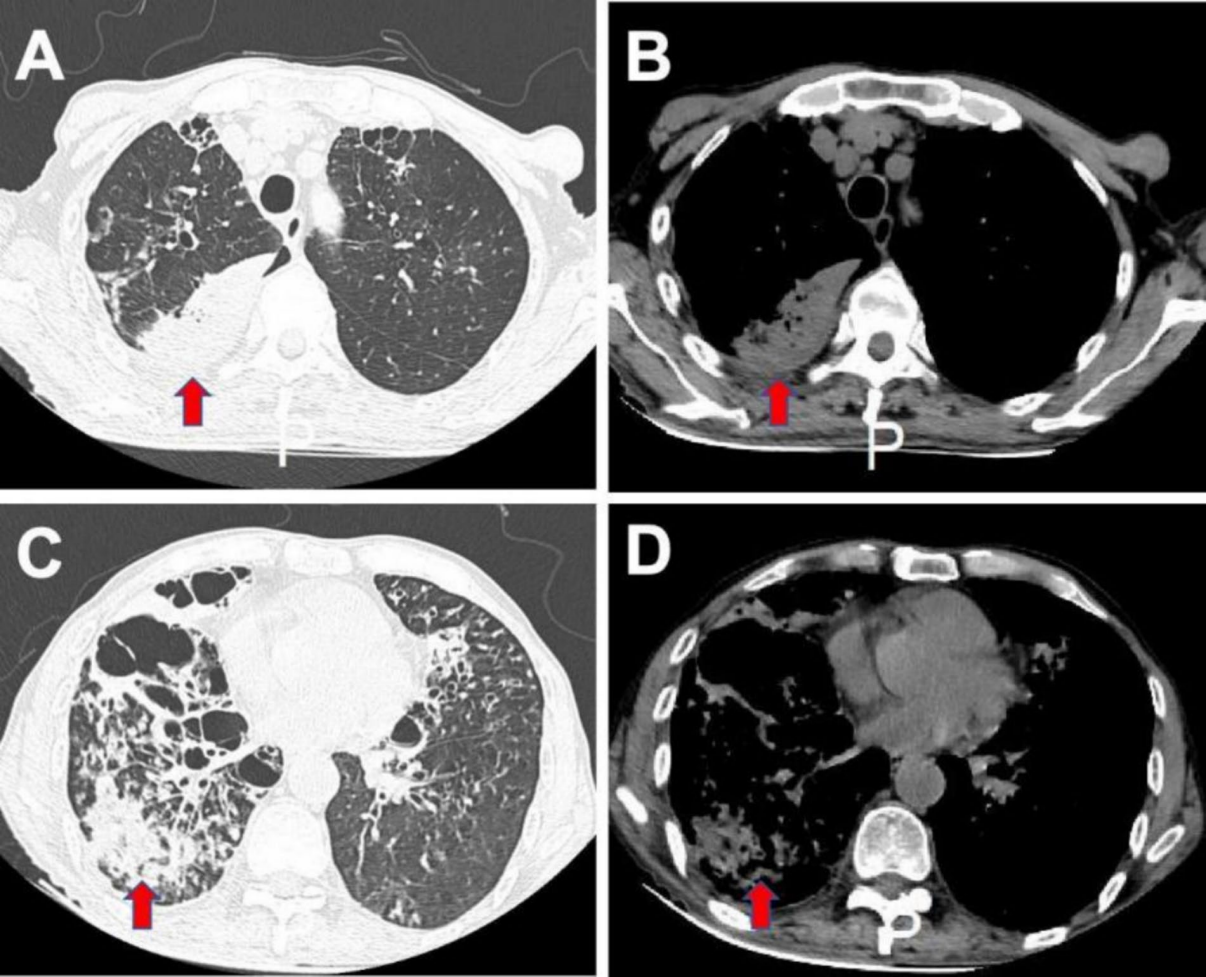
Chest CT scan before treatment. The CT scan showed bilateral bronchiectasis, with predominant involvement of the right lung, bronchial wall thickening, and multiple patchy opacities in both lungs. **(A,C)** Lung windows; **(B,D)** Mediastinal windows.

During the physical examination, the patient exhibited a fever of 38.2°C, and moist rales were auscultated over both lungs. Additionally, upon further examination, two carious teeth were discovered. Blood tests revealed elevated neutrophil levels (8.49 × 10^9/L), along with increased levels of C-reactive protein (CRP) at 215.7 mg/L and elevated procalcitonin (PCT) levels at 0.77 ng/mL. A chest CT scan illustrated multiple bilateral pulmonary bronchiectases with infection (Figure 1).

**Figure 2:**
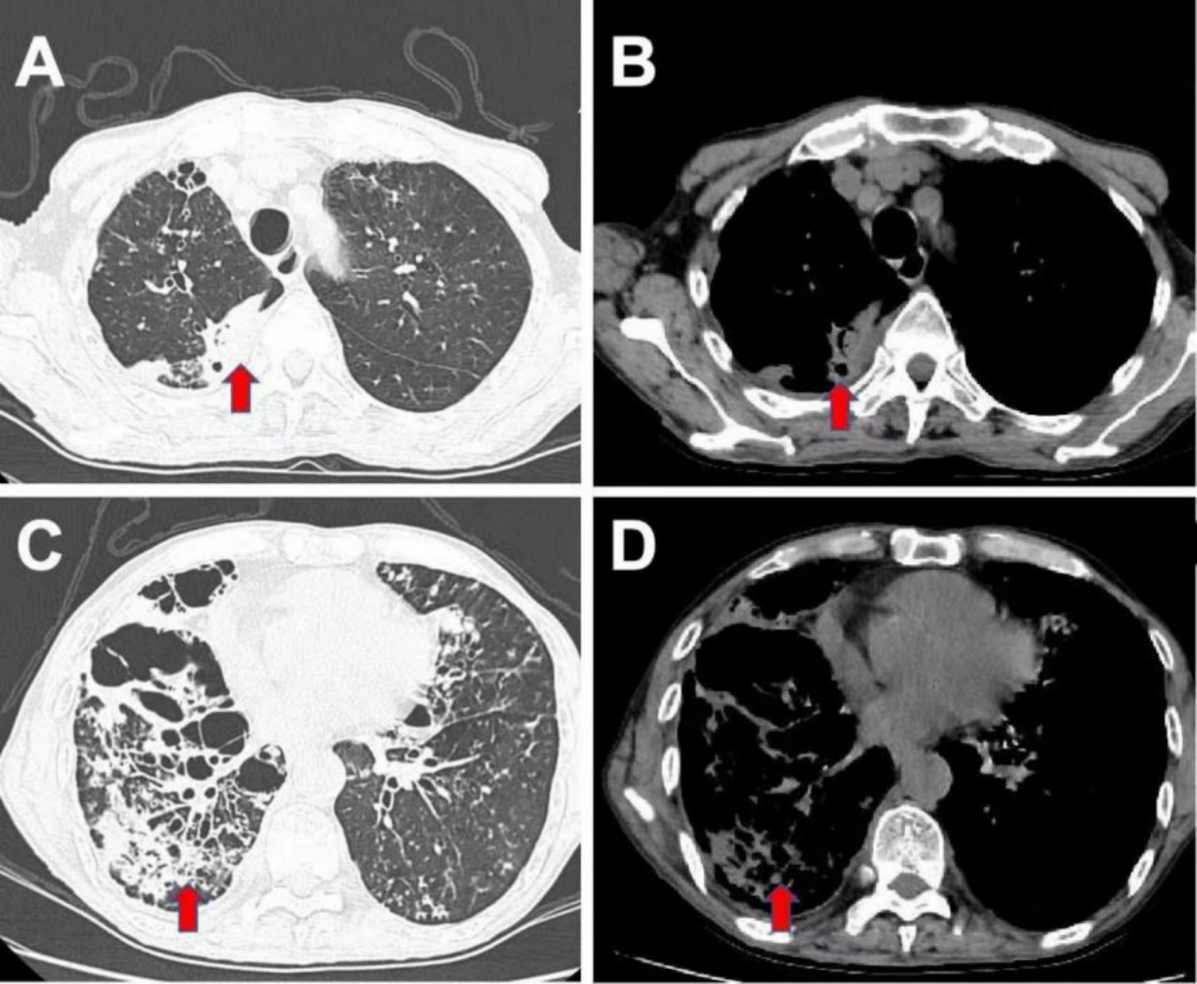
Chest CT scan after 10 days of anti-infection treatment. The CT scan showed similar findings of bilateral bronchiectasis and bronchial wall thickening, with a noticeable reduction in patchy consolidations. **(A,C)** Lung windows; **(B,D)** Mediastinal windows.

The patient was started on intravenous antibiotic therapy with piperacillin-tazobactam (4.5 g every 8 h) according to the guidelines of the British Thoracic Society (BTS) and the European Respiratory Society (ERS) (2, 3). However, after a week, the patient’s fever persisted, and serum CRP levels remained elevated at 111.8 mg/L. Subsequently, bronchoalveolar lavage was conducted to identify the causative pathogens. Metagenomic next-generation sequencing (mNGS) of the bronchoalveolar lavage fluid (BALF) identified multiple organisms including *Tannerella forsythia*, *Parvimonas micra*, *Fusobacterium nucleatum*, *Proteus mirabilis*, and *Streptococcus intermedius* (Table 1). No pathogens were isolated through conventional culture methods from the BALF. A dental examination was performed, revealing two dental caries in the patient. Although the patient denied a history of aspiration, we speculated that latent aspiration may be present. Consequently, treatment was later adjusted to combine piperacillin-tazobactam with ornidazole (0.5 g every 12 h, instead of metronidazole) for a 10-day course, following the guidelines of the European Federation of Periodontology (EFP) (4). Subsequent review of the chest CT scan (Figure 2) revealed partial resolution of the infection. The patient experienced alleviation of symptoms, and serum inflammatory markers showed overall normalization. Consequently, he was discharged from the hospital and prescribed oral antibiotics (amoxicillin and clavulanate potassium 312.5 mg tid) for an additional week.

**Table 1:** The result of mNGS in BALF.

Name of pathogen	Sequences
*Tannerella forsythia*	1965
*Parvimonas micra*	618
*Fusobacterium nucleatum*	208
*Proteus mirabilis*	38
*Streptococcus intermedius*	30

## Discussion

Bronchiectasis is a chronic pulmonary disease characterized by the dilatation of bronchi and persistent bacterial infection of the affected bronchial tubes (5). Individuals with bronchiectasis experience a higher frequency of annual respiratory tract infections and typically have a lower quality of life compared to healthy individuals (6). The most common bacteria associated with chronic bronchial infections include *Pseudomonas aeruginosa*, *Haemophilus influenzae*, and *Staphylococcus aureus* (7). In this report, we identified *Tannerella forsythia* infection in the BALF obtained from the patient’s diseased airways.

*Tannerella forsythia* is a kind of Gram-negative bacterium of Tannella, a common symbiotic bacteria in humans, and a member of the red complex of periodontal disease pathogens. The red complex oral bacteria includes *Porphyromonas gingivalis*, *Treponema denticola* and *Tannerella forsythia*, which always existing together. They are commonly found in oral cavity, associated with periodontitis, gingivitis and various cancers (8). They can inhibit monocyte chemotaxis by blocking the expression of adhesion molecules in intercelular reactions and block the body’s anti-inflammatory response (9). *Tannerella forsythia* has several virulence factors including proteases (KLIKK, PrtH), dipeptidyl peptidase IV, miropin, glycosidases (SusB, SiaHI, NanH, and HexA) and the OxyR protein (10). These virulence factors protect the bacterium from being killed in host tissue and promote disease progression. Although *Tannerella forsythia* infection is commonly associated with oral diseases, there is limited available literature on its involvement in lung infections. Recently, there have been two reports documenting *Tannerella forsythia* infection in cases of massive empyema and lung abscess (11, 12). Elderly patients with bronchiectasis are often weak and malnourished, frequently exhibiting impaired swallowing function and a potential for aspiration. Oral pathogens can reach the lower respiratory tract through aspiration, leading to infections. In some cases, patients with severe periodontitis may have oral pathogens enter the bloodstream, which can then infect the lungs. As in the oral cavity, *Tannerella forsythia* can damage the airway mucosa, facilitate the invasion of other pathogens, inhibit monocyte chemotaxis, and suppress the body’s anti-inflammatory response. In the present case, the patient had extensive bronchiectasis for many years and was found to be infected with *Tannerella forsythia* in the diseased airways.

*Tannerella forsythia* infection is frequently misdiagnosed due to the challenges associated with its detection using conventional culture techniques. Unlike many other bacteria, *Tannerella forsythia* lacks the biosynthetic pathways necessary for producing the sugar N-acetylmuramic acid (MurNAc). This characteristic may contribute to its low culture detection rate (13). In the present case, conventional culture methods failed to isolate any pathogens from the BALF, whereas mNGS identified *Tannerella forsythia*, along with other bacteria including *Parvimonas micra*, *Fusobacterium nucleatum*, *Proteus mirabilis*, and *Streptococcus intermedius*. Accurate microbiological identification is crucial for guiding appropriate clinical treatment. However, conventional methods are often time-consuming and may have a low detection rate. Conversely, mNGS demonstrates higher sensitivity in identifying pathogens and offers advantages in detecting potential pathogens (14). Zhang’s study found that mNGS demonstrated superior performance in detecting anaerobic or facultative anaerobic bacteria in patients with lung abscess compared to conventional culture methods, providing crucial evidence for clinical decision-making (15). The prompt application of mNGS benefits to short the time of diagnosism, enabling guided and accurate medication to reduce the risk of disease progression and reduce risk of death. Nonetheless, mNGS also has drawbacks, such as its high cost and the potential for generating data on multiple microorganisms, which can lead to confusion. Moreover, mNGS does not have a clear advantage in identifying fungi, viruses, and tuberculosis. In this case, we tailored the antibiotic therapy based on the results of mNGS, resulting in a favorable treatment outcome.

The antibiotic therapy to *Tannerella forsythia* infection is less reported. A study indicated that amoxicillin (AMX 500 mg tid for 14 days) plus metronidazole (MTZ 400 mg tid for 14 days) therapy were effective to reduct the red complex in patients with periodontitis (16, 17). While another report showed a failure of AMX + MTZ therapy to resolve peri-implantitis in 1 year follow-up (18). Metronidazole is highly active against anaerobic bacteria. Systemic metronidazole therapy can suppress red complex significantly. However, metronidazole was taken orally three times per day for 7–14 days, which needs patient compliance. Tinidazole is a second-generation 2-methyl-5-nitroimidazole class antibiotic structurally similar to metronidazole. Tinidazole exerts marked bactericidal activity against anaerobic bacteria and possesses pharmacokinetic properties which enables once-a-day oral systemic drug dosing (19). Ornidazole is a third-generation similar to tinidazole. Liu’s study found that the ecurative effect was no significant difference between ornidazole and mertonidazole, but the side effect of mertonidazole was more severe than that of ornidazole, especially irritaion of gastrointestinal tract (20). In this case, the patient had gastrointestinal discomfort after eating metronidazole tablets before and had no financial burden. Therefore, we chose the ornidazole this time. According to clinical guidelines, metronidazole is recommended as the first-line agent against Red Complex pathogens, while ornidazole may serve as a substitute option in specific cases.

It’s also reported that systemic Sitafloxacin (200 mg/day of STFX for 5 days) and Azithromycin (500 mg/day of AZM for 3 days) monotherapy significantly decreased red complex (21). A recent report showed that meropenem and moxifloxacin treatment were effective in lung abscess infected by *Tannerella forsythia* (12). Another case reported amoxicillin and amoxicillin-clavulanic acid treatment in a massive empyema pateint (11).

In this case, we chose piperacillin-tazobactam for an initial therapy and added ornidazole for a better therapeutic efficacy. As a kind of anaerobic bacteria, antibiotics to *Tannerella forsythia* infection are similar, but the specific antibiotic susceptibility of *Tannerella forsythia* needs more study to confirm.

## Conclusion

*Tannerella forsythia*, traditionally recognized as a member of the red complex of periodontal disease pathogens, has been increasingly implicated in pulmonary infections. In this case, we present the first documented instance of a patient with bronchiectasis being infected by *Tannerella forsythia*. Diagnosis of *Tannerella forsythia* infection is often reliant on mNGS, as conventional culture techniques frequently fail to confirm its presence. In our approach to treatment, we opted for a combination therapy of piperacillin-tazobactam and ornidazole, which yielded a favorable curative outcome. While antibiotics effective against anaerobic bacteria may demonstrate efficacy in treating *Tannerella forsythia* infection, further investigation into the specific antibiotic susceptibility of this pathogen is warranted. This case underscores the importance of considering unconventional pathogens in pulmonary infections and highlights the potential utility of mNGS in their identification and subsequent management.

## Data Availability

The original contributions presented in the study are included in the article/supplementary material, further inquiries can be directed to the corresponding author/s.
